# Musculoskeletal Disorders in Dentists: A Practical Review and Self-Management Framework

**DOI:** 10.1016/j.identj.2026.109792

**Published:** 2026-08-17

**Authors:** Volodymyr Maksym

**Affiliations:** Lviv State University of Physical Culture named after Ivan Boberskyi, Lviv, Ukraine

**Keywords:** Dentists, Musculoskeletal disorders, Ergonomics, Self-managment, Carpal tunnel syndrome, Exercise therapy

## Abstract

**Introduction and aims:**

Dentists work under sustained visual and manual demands that commonly require cervical flexion, forward trunk inclination, repetitive upper-limb activity and prolonged static sitting or standing. These exposures are associated with work-related musculoskeletal disorders (MSDs), particularly in the neck, back, shoulders and wrist-hand region. This review summarizes the burden, clinical pattern,and occupational drivers of MSDs in dentists and proposes a structured, evidence-informed self-management framework.

**Methods:**

A structured narrative review was conducted using PubMed/MEDLINE and citation tracking, with priority given to systematic reviews, meta-analyses, scoping reviews, randomized trials and key observational studies. Studies were eligible if they addressed the prevalence, anatomical distribution, occupational drivers, ergonomics, prevention, or conservative management of MSDs relevant to dental practice.

**Results:**

Dental-specific evidence consistently showed a high burden of MSDs in dentists. A meta-analysis reported a pooled overall MSD prevalence of 78.4%, while pooled regional prevalence estimates in Western dental professionals were 58.5% for the neck, 56.4% for the lower back, 43.1% for the shoulder, and 41.1% for the upper back. Carpal tunnel syndrome was also clinically relevant, with pooled prevalence estimates ranging from 9.87% in dentists to 15% in broader dental health care personnel. Dental-specific intervention evidence most strongly supported ergonomic optimisation, visual magnification and workplace ergonomic modification. Direct evidence for dentist-specific exercise-based protocols remained limited, whereas broader rehabilitation literature supported exercise-based care for chronic spinal pain and adjunctive conservative approaches for selected mild wrist-hand symptoms. Lower-limb and pedal-related complaints appeared plausible but under-investigated.

**Conclusion:**

Dentists represent a high-risk occupational group for MSDs. A structured, evidence-informed framework that prioritises ergonomic optimisation and adds feasible self-management strategies may assist prevention and early symptom management in routine dental practice.

**Clinical Relevance:**

The proposed framework organises current evidence into a practical and low-cost model that may be implemented in routine dental settings without specialised equipment.

## Introduction

Dentistry is a precision profession, but it is also a physically demanding one. During clinical work, dentists commonly maintain forward head posture, cervical flexion, trunk inclination, shoulder elevation and prolonged sitting or standing while performing repetitive fine-motor tasks.^1^, ^2^, ^3^, ^4^ These exposures are repeated across patients, working days and years of practice. The result is a substantial burden of work-related musculoskeletal disorders (MSDs), particularly in the neck, lower back, shoulders, upper back and wrist-hand region.^1^, ^2^, ^3^, ^4^

This burden matters clinically and professionally. Dentists rely on sustained concentration, stable posture and manual precision. Even moderate recurrent pain may therefore impair comfort, work ability and productivity. In a study of working dentists, decreased productivity was associated with musculoskeletal pain, poor sleep and stress.^5^ MSDs in dentistry are therefore not a minor occupational inconvenience, but a meaningful threat to career sustainability.

At the same time, an important practical gap remains. Dental-specific reviews consistently support ergonomic measures, yet high-quality evidence for exercise-based prevention and rehabilitation in dentists is still limited.^6^, ^7^, ^8^ Clinicians therefore need realistic guidance before the literature becomes ideal.

The aim of this concise review is twofold: first, to summarise the burden, clinical pattern, and occupational drivers of MSDs in dentists; and second, to propose a pragmatic, evidence-informed self-management framework for prevention and early symptom control. This framework is not intended as a formal guideline or a substitute for individualised care. Rather, it is a practical structure that integrates dental-specific ergonomic evidence with broader rehabilitation evidence for common spinal and upper-limb disorders relevant to dental practice.

## Methods

### Review design

This revised manuscript was prepared as a structured narrative review designed to summarise the burden, anatomical distribution, occupational drivers, and relevant self-management implications of work-related musculoskeletal disorders (MSDs) in dentists.

### Search strategy

Electronic searches were conducted in PubMed/MEDLINE up to 03/07/2026, supplemented by backward citation tracking of key reviews and included studies. Additional relevant studies were identified through backward citation tracking of key reviews and included articles.

The PubMed search strategy combined terms related to dental practice and musculoskeletal disorders/interventions, as follows:

(“dentist” OR “dental professional” OR “dental personnel” OR “oral health professional” OR “dental hygienist”) AND (“musculoskeletal disorder” OR “musculoskeletal pain” OR “ergonomics” OR “posture” OR “neck pain” OR “back pain” OR “low back pain” OR “shoulder” OR “wrist” OR “hand” OR “carpal tunnel syndrome” OR “rehabilitation” OR “exercise” OR “stretching” OR “microbreak”)

The Scopus search used the same conceptual combination of terms adapted to database syntax.

### Eligibility criteria

Studies were considered eligible if they met the following criteria:

1. included dentists, dental professionals, or oral health personnel as the study population;
2. addressed the prevalence, anatomical distribution, occupational risk factors, ergonomics, prevention, rehabilitation, or conservative management of MSDs relevant to dental practice;
3. were published in English;
4. were systematic reviews, meta-analyses, scoping reviews, randomised controlled trials, controlled clinical studies, cohort studies, cross-sectional studies, or other observational studies with clear methodological description.

Studies were excluded if they:

1. focused on non-dental occupations without clearly transferable relevance to dental practice;
2. did not address musculoskeletal outcomes or occupational physical loading;
3. were editorials, opinion pieces, narrative essays without identifiable methods, conference abstracts without sufficient data or duplicate publications;
4. lacked sufficient information to extract study focus and main findings.

### Study selection

Titles and abstracts were screened for relevance by the author. Full texts were then assessed against the eligibility criteria. Where more than 1 report described the same dataset or substantially overlapping findings, the most informative or methodologically complete source was retained.

Relevant records were identified through database searching and backward citation tracking. Titles and abstracts were screened for relevance, followed by full-text assessment against the eligibility criteria. Studies meeting the inclusion criteria were included in the final synthesis.

### Data extraction

For each included study, the following information was extracted: author and year, country, study design, study population, main anatomical region or MSD focus, occupational context or exposure of interest, and principal findings relevant to prevalence, risk factors, ergonomics or self-management implications.

### Data synthesis

Because the included studies were heterogeneous in design, populations, outcomes, and intervention characteristics, a quantitative synthesis was not attempted. Instead, findings were synthesised narratively and organised into the following clinically relevant domains:

1. burden and anatomical pattern of MSDs in dentists;
2. occupational drivers and ergonomic risk factors;
3. wrist-hand disorders and carpal tunnel syndrome;
4. lower-limb symptoms and pedal-related loading;
5. implications for a practical, evidence-informed self-management framework.

## Results

### Burden and clinical pattern of MSDs in dentists

The included dental-specific evidence consistently showed that musculoskeletal disorders (MSDs) are highly prevalent in dental professionals, with the neck, lower back, shoulders, and upper back representing the most commonly affected regions.^1^, ^2^, ^3^, ^4^ Across reviews, the epidemiological pattern was reproducible despite variation in study design, country and case definition.^1^, ^2^, ^3^, ^4^ The wrist-hand region also emerged as a clinically important burden area, particularly in the context of repetitive instrumentation and sustained fine-motor work.^4^^,^^9^^,^^10^

A summary of the key dental-specific studies included in this review is presented in Table 1.

**Table 1:** Key dental-specific studies included in the review.

Study	Design / population	Main focus	Key findings	Relevance to this review
Chenna et al.^1^	Systematic review and meta-analysis; dental healthcare providers	Overall MSD prevalence	Showed a high pooled prevalence of MSDs in dental personnel	Establishes the overall burden of MSDs in dentistry
Lietz et al.^2^	Systematic review and meta-analysis; dental professionals in Western countries	Prevalence and risk factors	Neck, lower back, shoulder, and upper back were most affected; posture, repetition, and workload were major risk factors	Supports the core anatomical and ergonomic focus of the review
Soo et al.^3^	Systematic review; dentists	Ergonomics and MSDs	Confirmed the strong association between poor ergonomics and MSDs in dentists	Supports the ergonomics-first principle
Jain et al.^4^	Umbrella review; dental professionals	MSD patterns across reviews	Reinforced the burden in neck, back, shoulder, and wrist-hand regions; lower-limb symptoms were less studied	Supports inclusion of wrist-hand symptoms and cautious discussion of lower-limb issues
Roll et al.^6^	Systematic review; oral health care professionals	Prevention and rehabilitation	Found strongest support for ergonomic adaptation and workplace-focused preventive strategies	Supports ergonomic optimisation as the main first-line intervention
Wu et al.^7^	Scoping review; dental professionals	Prevention of work-related MSDs	Showed that dental literature emphasises ergonomics more than exercise-based prevention	Justifies combining dental-specific evidence with broader rehabilitation evidence
Lin et al.^8^	Cluster-randomised controlled trial; young dental professionals	Ergonomic intervention	Participatory ergonomic intervention reduced risk exposure and improved selected outcomes	Provides direct interventional support for workplace ergonomics
Chenna et al.^9^	Systematic review and meta-analysis; dental health care personnel	Carpal tunnel syndrome	Showed that CTS is sufficiently prevalent in dental personnel to warrant focused attention	Supports inclusion of a wrist-hand module
Kostares et al.^10^	Systematic review and meta-analysis; dentists	Carpal tunnel syndrome in dentists	Confirmed that CTS is not rare among dentists	Strengthens the wrist-hand/entrapment branch of the review

### Occupational drivers and ergonomic factors

The reviewed dental literature consistently identified awkward posture, prolonged static loading, repetitive upper-limb activity, high patient volume, insufficient breaks and visual-motor constraints as the principal occupational drivers of MSDs in dentistry.^2^, ^3^, ^4^ These factors help explain why symptoms tend to cluster in the cervical, thoracic, lumbar, shoulder and wrist-hand regions.^2^, ^3^, ^4^

The interventional literature was more limited than the epidemiological literature but supported an ergonomics-first approach. Reviews and scoping evidence indicated that magnification, indirect-vision techniques and structured workplace ergonomic modification were among the most consistently supported preventive strategies in dental settings.^6^, ^7^, ^8^ A cluster-randomised trial further suggested that participatory ergonomic intervention can reduce ergonomic risk exposure and improve selected neck and wrist-hand outcomes in young dental professionals.^8^

### Wrist-hand disorders and carpal tunnel syndrome

The distal upper limb deserves explicit consideration in dentists. In addition to general wrist-hand discomfort, meta-analytic evidence indicated that carpal tunnel syndrome (CTS) is not rare in dental personnel.^9^^,^^10^ Taken together, these findings support treating wrist-hand symptoms and early entrapment neuropathies as a core clinical domain in dental occupational health rather than as a secondary extension of general MSD burden.

### Lower-limb symptoms and the pedal question

Compared with spinal and upper-limb symptoms, lower-limb complaints remain less well studied in dentistry. Nevertheless, umbrella-level evidence suggests that symptoms in the hips, thighs, knees, legs, ankles, and feet are present in at least a subgroup of dental professionals.^4^ Direct evidence linking these complaints specifically to sustained foot-pedal operation remains limited. However, observational data showing asymmetrical plantar pressure patterns in dental personnel support the plausibility of lower-limb loading asymmetry as a relevant but under-investigated occupational issue.^11^

### Implications for framework development

Overall, the evidence reviewed in dental populations provided strong support for the burden of MSDs and for the importance of ergonomic risk reduction, but more limited direct support for dentist-specific exercise-based or self-management protocols.^1^, ^2^, ^3^, ^4^, ^5^, ^6^, ^7^, ^8^ This pattern justified the present translational approach: a framework grounded first in dental-specific evidence on burden and ergonomics, and second in broader rehabilitation evidence for chronic spinal pain and mild upper-limb entrapment disorders.

## Discussion

### Translating the evidence into a practical framework

The present review highlights a familiar but important pattern in dental occupational health: the epidemiological burden of musculoskeletal disorders (MSDs) in dentists is well supported, whereas the evidence base for dentist-specific self-management or rehabilitation protocols remains comparatively limited.^1^, ^2^, ^3^, ^4^, ^5^, ^6^, ^7^, ^8^ The literature is therefore stronger in defining the problem than in prescribing a complete solution. This imbalance shaped the present framework.

**Fig. 1:**
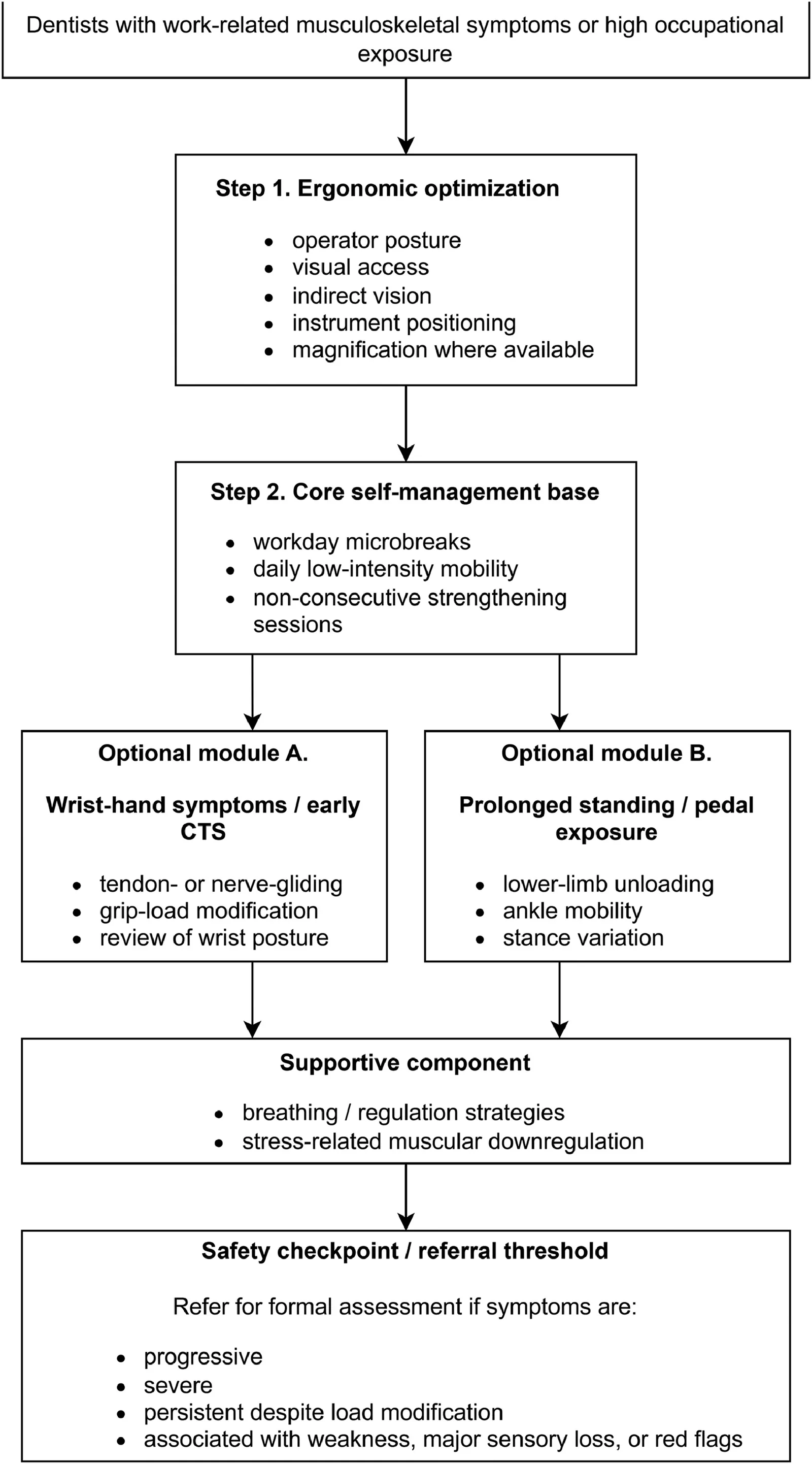
Proposed evidence-informed self-management framework for dentists.

Rather than presenting a formal guideline, this review proposes a structured, evidence-informed framework that integrates 2 levels of evidence. The first level is dental-specific and supports the high burden of MSDs in dentists, the anatomical concentration of symptoms in the neck, back, shoulders, and wrist-hand region, and the importance of ergonomic risk reduction.^1^, ^2^, ^3^, ^4^, ^5^, ^6^, ^7^, ^8^, ^9^, ^10^ The second level is broader rehabilitation evidence, which supports exercise-based management for chronic spinal pain and adjunctive conservative approaches for selected mild upper-limb entrapment symptoms.^12^, ^13^, ^14^, ^15^, ^16^ The proposed framework is summarised in Table 2 and Figure 1.

**Table 2:** Proposed evidence-informed self-management framework for dentists.

Framework component	Target problem / body region	Evidence base	Suggested implementation in dental practice	Cautions / limits
Ergonomic optimisation	Global MSD risk; neck, back, shoulders, wrist-hand region	Direct dental-specific evidence	Optimise operator position, visual access, indirect vision, instrument positioning, and use of magnification where available; prioritise ergonomic correction before adding self-management strategies	Should not be replaced by exercise alone; workplace correction remains the first-line measure
Workday microbreaks	Static loading; neck, shoulders, low back, gripping hand	Direct dental-specific rationale; indirect supportive occupational evidence	Brief postural reset between patients or when workflow permits; change spinal position, relax shoulders, unload the dominant hand, vary stance, and restore neutral posture	Feasibility depends on patient flow and clinical setting; not a substitute for broader ergonomic correction
Daily low-intensity mobility block	Stiffness; postural fatigue; neck, thoracic spine, low back, shoulders	Indirect broader rehabilitation evidence; clinically plausible dental application	Short daily mobility routine performed once daily, preferably after clinical work or during non-clinical time; may include spinal mobility, thoracic rotation/extension, shoulder opening, scapular reset, gentle wrist/forearm mobility, and lower-limb unloading movements	Should be low-load and non-fatiguing; presented as a practical adjunct, not a validated dentist-specific protocol
Non-consecutive strengthening sessions	Reduced load tolerance; recurrent spinal and shoulder symptoms	Indirect broader rehabilitation evidence for chronic low back and neck pain	Strengthening and motor-control work 2-3 times weekly on non-consecutive days; emphasise trunk endurance, scapular control, hip/trunk stability, and posterior-chain capacity	Exercise selection should remain principle-based rather than prescriptive; avoid symptom aggravation and excessive soreness that interferes with clinical work
Wrist-hand symptom module	Wrist-hand discomfort; early overuse symptoms; mild CTS	Dental-specific prevalence evidence; indirect conservative CTS evidence	For clinicians with intermittent or early symptoms, consider tendon-gliding or nerve-gliding, grip-load modification, symptom-limited forearm mobility, and review of wrist posture during instrumentation	Not appropriate for progressive numbness, objective weakness, persistent night symptoms, thenar weakness or major functional loss; these require formal assessment
Lower-limb unloading module	Prolonged standing; asymmetrical stance; possible pedal-related loading	Limited dental-specific evidence; exploratory/indirect support	Optional module for dentists with prolonged standing or sustained pedal use; may include ankle mobility, heel-toe shifting, calf work, brief hip-extension movements, and lower-limb unloading after sessions	Should be presented as preventive and hypothesis-generating; current evidence does not establish a specific pedal-related syndrome
Breathing / regulation strategies	Stress-related muscular overactivity; symptom amplification; recovery support	Limited indirect evidence outside dental populations	Brief diaphragmatic breathing, paced breathing, or short regulation practice may be added as a supportive element, especially during periods of high stress	Supportive only; should not be presented as a core MSD treatment or as equivalent to ergonomic and exercise-based strategies
Red flags / referral threshold	Safety across all modules	Clinical safety principle	Refer for medical or rehabilitation assessment when symptoms are progressive, severe, persistent despite load modification, or suggest neurological, inflammatory, or systemic pathology	Self-management is for prevention, early symptoms, and non-complicated recurrent complaints, not for unexplained or progressive pathology

This distinction is important for scientific transparency. The framework should not be interpreted as a validated dentist-specific treatment protocol. Rather, it is a translational model derived from the best currently available dental evidence, supplemented where necessary by higher-level rehabilitation literature from adjacent musculoskeletal fields.

### Ergonomic optimisation as the foundation

The strongest intervention message emerging from the dental literature is that ergonomics should remain the foundation of any preventive or self-management strategy. Reviews focused on oral health professionals consistently support measures such as improved operator positioning, visual access, indirect-vision techniques, visual magnification, and broader workplace ergonomic modification.^6^, ^7^, ^8^ The cluster-randomised study by Lin et al. further suggests that structured ergonomic intervention can improve selected outcomes related to MSD burden and work ability in dental settings.^8^ Recent interventional evidence also suggests that technology-assisted ergonomics training may be useful in dental education. In a randomised study of 52 dental students from 3 universities in South Korea, virtual reality-based real-time ergonomics training reduced high-risk postures and improved ergonomic awareness, although some regression was observed after the intervention period.^12^

For this reason, the framework proposed here is organised hierarchically, with ergonomic optimisation as the first step rather than as 1 component among equals. Exercise, mobility, and symptom-specific self-management may support dentists, but they should not be expected to compensate for a poorly organised or persistently aggravating work environment.

### Core daily self-management strategies

After ergonomic correction, the next layer of the framework consists of core strategies intended to reduce uninterrupted physical load and improve tolerance to routine clinical work. These include workday microbreaks, a brief daily low-intensity mobility block and non-consecutive strengthening sessions.

Microbreaks are included because the reviewed dental literature repeatedly identifies static loading, constrained posture, and repetition as central occupational exposures.^2^, ^3^, ^4^ Although direct trial evidence for precise microbreak dosing in dentists remains limited, the concept is operationally relevant to clinical practice: even short opportunities to vary spinal position, relax the shoulders, unload the dominant hand, or change stance may reduce uninterrupted exposure to low-amplitude static loading.

The daily low-intensity mobility component is presented more cautiously. It is not based on a validated dentist-specific protocol and it should not be interpreted as such. Rather, it reflects a practical application of broader rehabilitation principles to a profession characterised by repetitive postural strain. Its role in the framework is to promote movement variability, reduce stiffness after static work, and support body awareness without creating additional fatigue that might interfere with clinical performance.

The strengthening component is supported more strongly by broader rehabilitation literature than by dental-specific trials. Exercise therapy has demonstrated benefit in chronic low back pain,12 while guideline-level evidence also supports non-pharmacological and exercise-based approaches for neck and low back pain.^14^ Network meta-analytic evidence in chronic non-specific neck pain suggests that several exercise approaches may be effective, without a single universally superior method.^15^ This supports a principle-based rather than method-based prescription. In other words, the framework does not claim that one named exercise is uniquely appropriate for dentists; rather, it supports regular, tolerable strengthening and motor-control work that improves load tolerance in clinically relevant regions.

### Wrist-hand symptoms and early entrapment complaints

One of the clearest ways in which the present review extends beyond a generic spinal discussion is by explicitly identifying wrist-hand symptoms and carpal tunnel syndrome (CTS) as central occupational issues in dentists.^4^^,^^9^^,^^10^ This matters because dental work combines repetitive instrumentation, sustained grip activity, and non-neutral wrist loading, all of which are relevant to distal upper-limb strain.^6^

Accordingly, the framework includes a specific wrist-hand module, but this must be interpreted carefully. It is intended for clinicians with intermittent or early symptoms, such as transient paraesthesia, activity-related discomfort, mild symptom fluctuation, or early hand/wrist irritation without progressive weakness, thenar atrophy, persistent sensory deficit or major functional loss. In such cases, tendon-gliding or nerve-gliding, review of wrist posture during instrumentation, and grip-load modification may be considered as adjunctive conservative measures.^16^^,^^17^

However, this part of the framework has clearer safety boundaries than the spinal components. Self-management is not sufficient when symptoms are progressive, nocturnal and worsening, associated with objective weakness, accompanied by persistent numbness or suggest more advanced neuropathy. In those cases, formal medical or rehabilitation assessment is more appropriate. The purpose of including this module is therefore not to encourage unsupervised management of established neuropathy, but to acknowledge that wrist-hand complaints are common in dentists and deserve structured early attention.

### Lower-limb symptoms and pedal-related exposure

Lower-limb symptoms remain an underdeveloped area in the dental MSD literature. The umbrella-level evidence suggests that symptoms in the hips, thighs, knees, legs, ankles and feet do occur in dental professionals, but they have not been characterised with the same consistency as cervical, lumbar, shoulder or wrist-hand complaints.^4^ Direct evidence linking these symptoms specifically to prolonged foot-pedal use is still limited.

For that reason, the lower-limb module in the framework is deliberately cautious. It is presented as an exploratory and preventive component rather than as a validated response to a clearly established pedal-related syndrome. The inclusion of lower-limb unloading, stance variation and ankle mobility is justified not by strong dental intervention trials, but by the plausibility of asymmetrical loading in prolonged standing work and by observational evidence suggesting altered plantar pressure patterns in dental personnel.^11^ This area should be regarded as hypothesis-generating and in need of further targeted study.

### Supportive regulation strategies

Breathing and regulation strategies occupy a secondary place in the proposed framework. They are included not because they are equivalent to ergonomic correction or exercise-based management, but because dental work combines physical strain with sustained cognitive and emotional demand.^5^ In some clinicians, stress-related muscular overactivity may plausibly amplify symptom persistence or reduce recovery.

At the same time, the evidence supporting this component is indirect and not dental-specific. Reviews of mindfulness-based interventions for chronic low back pain and breathing-based interventions for persistent neck pain suggest potential benefit, but certainty remains limited and the populations studied are not specific to dentistry.^18^^,^^19^ For this reason, regulation strategies should be viewed as supportive rather than foundational. They may be useful for symptom modulation, body awareness and stress management, but they should not be overinterpreted as primary treatment for work-related MSDs in dentists.

### Clinical implications and implementation logic

A major criticism of many broad occupational health recommendations is that they are correct in principle but difficult to operationalise. The present framework attempts to address that gap by organising interventions in a layered way. Ergonomic optimisation forms the foundation, followed by core daily strategies that are feasible within routine clinical work, and then by optional modules for selected symptom patterns. This structure is intended to improve usability in real-world dental settings.

Importantly, the framework is also designed to remain low-cost and portable. It does not depend on specialised exercise equipment, extensive treatment time or access to formal rehabilitation facilities. This feature does not prove effectiveness, but it increases feasibility across diverse dental settings and may support adherence better than more resource-intensive models.

### Limits of self-management and implications for future research

The present framework also has clear limitations. It is not a systematic review with formal risk-of-bias assessment, and it is not a clinical guideline developed through consensus methods. Several of its components – especially those related to exercise dosing, lower-limb unloading, and supportive regulation strategies – are based on translational reasoning from broader rehabilitation evidence rather than on multiple dentist-specific randomised trials. This should be viewed as a limitation of the current evidence base, not as proof that such components are ineffective.

Future research should move beyond prevalence description and test implementation-focused interventions in practicing dentists. Specifically, studies are needed to determine whether a layered model combining ergonomic correction, microbreaks, mobility, strengthening, and symptom-specific modules can improve pain, function, work ability and productivity in dental practice. The lower-limb and pedal-related branch appears especially in need of targeted investigation one.

## Conclusions

Musculoskeletal disorders are a major occupational health concern in dentistry, with the neck, lower back, shoulders, upper back and wrist-hand region representing the most consistently affected anatomical areas. Current dental-specific evidence strongly supports the burden of these disorders and the importance of ergonomic risk reduction, while direct evidence for dentist-specific self-management and rehabilitation protocols remains more limited.

The present review therefore proposes a structured, evidence-informed framework that places ergonomic optimisation at its foundation and adds feasible self-management strategies, including microbreaks, low-intensity mobility, non-consecutive strengthening, and selected symptom-specific modules. Wrist-hand symptoms and carpal tunnel syndrome deserve explicit attention in dentists, whereas lower-limb and pedal-related complaints remain plausible but under-investigated.

This framework should be interpreted as a translational and practice-oriented model rather than a validated clinical protocol. Its main value lies in organising current evidence into a practical structure that may assist prevention and early symptom management in routine dental practice. Future studies should test whether such a layered approach can improve pain, function, work ability and productivity in dentists.

## Author contributions

*Concept and design:* Volodymyr Maksym.

*Literature search, study selection, data extraction, analysis, and interpretation:* Volodymyr Maksym.

*Drafting of the manuscript:* Volodymyr Maksym.

*Critical revision of the manuscript for important intellectual content:* Volodymyr Maksym.

## Funding

This research did not receive any specific grant from funding agencies in the public, commercial or not-for-profit sectors.

## Declaration of generative AI and AI-assisted technologies in the manuscript preparation process

During the preparation of this manuscript, the author used generative AI tools to assist with language refinement, text editing, and organisational support during drafting and revision. All literature selection, interpretation of evidence, critical revision, and final approval of the manuscript were performed by the author, who takes full responsibility for the content of the work.

## Declaration of competing interest

None disclosed.
